# A homozygous truncating variant in *CCDC186* in an individual with epileptic encephalopathy

**DOI:** 10.1002/acn3.51260

**Published:** 2020-12-01

**Authors:** Melanie Brugger, Fiona Becker‐Dettling, Theresa Brunet, Tim Strom, Thomas Meitinger, Eberhard Lurz, Ingo Borggraefe, Matias Wagner

**Affiliations:** ^1^ Institute of Human Genetics School of Medicine Technical University of Munich Munich Germany; ^2^ Division of Pediatric Neurology, Developmental Medicine and Social Pediatrics Department of Pediatrics Dr. von Haunersches Childrens Hospital Ludwig Maximilians University of Munich Munich Germany; ^3^ Division of Pediatric Gastroenterology Dr. von Haunersches Childrens Hospital Ludwig Maximilians University of Munich Munich Germany; ^4^ Comprensive Epilepsy Center Ludwig Maximilians University of Munich Munich Germany; ^5^ Institute of Neurogenomics Helmholtz Zentrum München GmbH German Research Center for Environmental Health Neuherberg Germany

## Abstract

Coiled‐Coil Domain Containing Protein 186 (CCDC186) is hypothesized to play an important role in the biogenesis of dense‐core vesicles in neurons and endocrine cells. Biallelic loss‐of‐function variants in the encoding gene *CCDC186* have been suggested as a candidate gene for a neurodevelopmental phenotype, but only one patient has been described so far. We report a second patient with a *CCDC186*‐associated phenotype presenting with developmental delay, epileptic encephalopathy, and failure to thrive. Exome sequencing identified a homozygous loss‐of‐function variant in *CCDC186* (NM_018017.2) c.767C> G; p.(Ser256Ter) thus providing further evidence to support *CCDC186* as a new disease gene for an autosomal recessive neurodevelopmental disorder.

## Introduction

**Figure 1:**
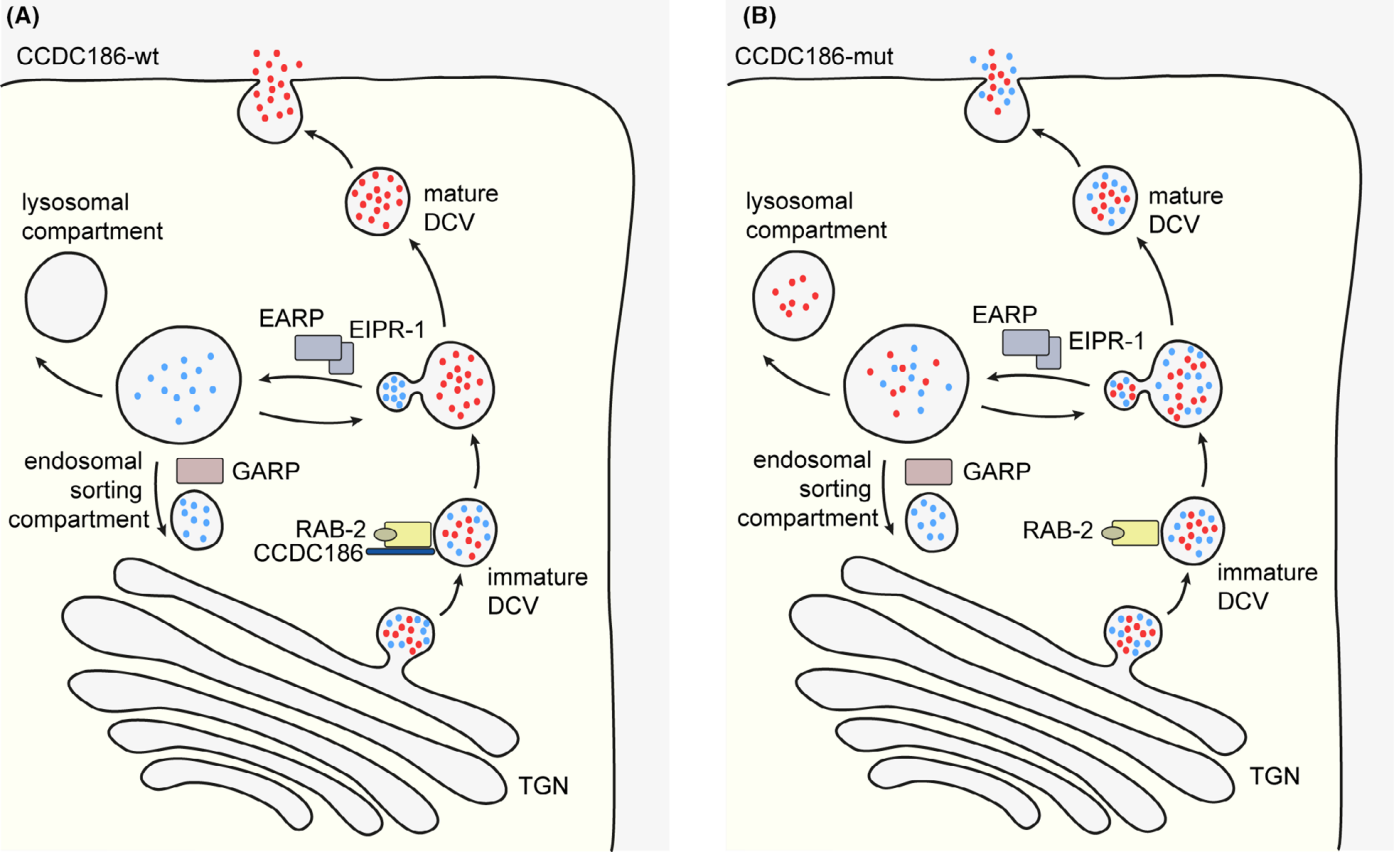
Simplified illustration of biogenesis of dense‐core vesicles (DCVs) and cargo sorting in CCDC186‐wildtype (wt) and CCDC186‐deficient (mut) cells. (A): Immature DCVs with soluble cargo such as peptides and monoamines (depicted as red and blue dots) are generated at the trans Golgi network (TGN). Cargo sorting after vesicle formation is mediated by the active, GTP‐bound RAB‐2 and CCDC186, among other. Following cargo sorting, several maturation steps including acidification and processing of cargo leads to mature DCVs, which are stored until release upon stimulation of the cell. DCV cargo not destined for secretion is transported to the endosomal sorting compartment with help of the endosome associated recycling protein (EARP) complex and EARP inhibitor protein EIPR‐1. From the endosomal compartment, cargo can be either shuttled back to the TGN via the Golgi associated recycling protein (GARP) complex or processed for lysosomal degradation. (B) Cells deficient of CCDC186 are predicted to have impaired cargo sorting in DCVs, resulting in secretion of incorrectly sorted cargo. Additionally, cargo might be falsely steered towards lysosomal degradation thus leading to a reduced concentration of the secreted cargo.

Coiled‐Coil Domain Containing 186 (CCDC186) is a membrane‐associated protein encoded by the *CCDC186* gene. Its orthologue in *C. elegans* CCCP‐1 was recently found to participate in secretory dense‐core vesicle (DCV) trafficking, possibly affecting maturation, cargo sorting, and tethering of vesicles (see Fig. 1)^1^, ^2^, ^3^. In contrast to neurotransmitters targeting ion channels that are released from synaptic vesicles, neuromodulators of the nervous system are secreted from DCVs generated at the trans‐Golgi network (TGN). Neuronal DCVs, which are presumably equivalent to secretory vesicles in (neuro)‐endocrine and exocrine cells, contain signaling molecules thus modulating various intra‐ and intercellular processes.^4^, ^5^ CCCP‐1 was established as a downstream effector of RAB‐2, one of many Rab GTPases involved in DCV trafficking.^1^, ^2^, ^6^ Additionally, CCCP‐1/CCDC186 co‐localizes with the endosome‐associated recycling protein (EARP) protein complex and the EARP interactor EIPR‐1, which are both responsible for sorting and recycling of cargo via the endosomal compartment.^7^

The orchestrated release of neuromodulators is essential for proper development and function of the nervous system. Variants in genes encoding for proteins involved in synaptic vesicular trafficking are increasingly recognized as a cause of neurodevelopmental disorders.^8^ The importance of DCV trafficking in brain development is underlined by the fact that two protein members of the EARP complex, *VPS‐51* and *VPS‐53,* have been associated with neurodevelopmental disorders.^9^, ^10^, ^11^, ^12^ To date, an association of biallelic loss‐of‐function variants in *CCDC186* with a neurodevelopmental disorder has been proposed and described in a single patient.^13^ We report a second pediatric patient with a *CCDC186*‐associated phenotype comprising of failure to thrive, developmental delay and medically refractory epilepsy, providing further evidence for a disease association of *CCDC186* variants.

## Patients and Methods

### Probands and samples

Clinical, laboratory, metabolic, and neuroradiologic data were acquired at the Department of Pediatrics (Dr. von Haunersches Kinderspital) at the University Hospital of Munich, Germany. Repeated 24‐channel electroencephalography (EEG) recordings were performed using standard adjustments. Magnetic resonance imaging (MRI) of the brain was obtained using a 3‐T high‐resolution scanner.

### Exome sequencing

Exome sequencing (ES) was performed in the frameworks of the German health care project “TRANSLATE‐NAMSE.” The legal guardians of the patient were included in a study that was approved by the local Ethics Committee of the Technical University of Munich and gave written informed consent for genetic studies and the publication of findings.

ES of the index patient and her parents was performed as previously described.^14^ Sure Select Human All Exon 60 Mb V6 kits (Agilent, Santa Clara, CA, USA) were used for the library preparation and enrichment of coding regions. Sequencing was performed on an Illumina NovaSeq 6000 sequencer (Illumina, San Diego, CA, USA). The BWA algorithm v.0.5.9. was used to align reads to the UCSC human reference assembly (hg19). Average read‐depth was more than 103‐fold and more than 96% of exons were covered at least 20‐fold. Single‐nucleotide variants (SNVs) and small insertions and deletions were detected by SAMtools v.0.1.19. The software ExomeDepth was applied to call copy number variations (CNVs). In‐house custom Perl scripts were used for variant annotation.

## Results

### Clinical findings

The 15 months old female patient was born small for gestational age (birth weight 2450g) after 37 + 1 gestational weeks as the first child of consanguineous Senegalese parents with normal postnatal adaptation but congenital pulmonary artery stenosis. No distinct facial or dysmorphic features were noted.

**Figure 2:**
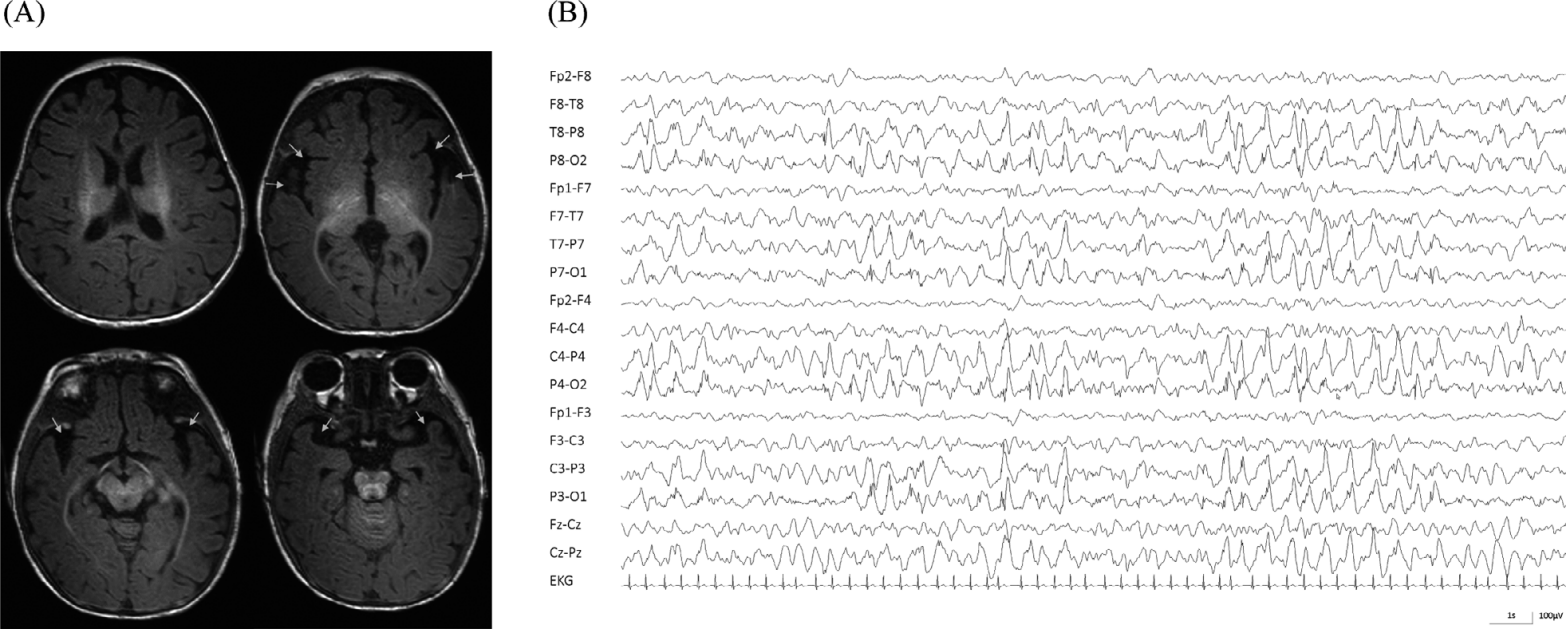
(A) Sleep EEG at the age of 12 months in a standard bipolar montage reveals severe epileptic encephalopathic pattern with predominant bilateral posterior slowing and continuous interictal spikes resembling hypsarrhythmia. There is lack of normal sleep architecture with absence of vertex waves and sleep spindles. (B) T1 weighted MRI images showing frontotemporal atrophy (arrows) of our patient at the age of 5 months.

First assessments at the age of 4 months revealed global developmental delay and muscular hypotonia. She developed seizures and was successfully treated with levetiracetam. At the age of five months, a brain MRI showed unspecific frontotemporal atrophy (Fig. 2A). At the age of 7 months, severe epileptic encephalopathy (West‐Syndrome) was present and both seizures (epileptic spasms) and EEG (hypsarrhythmia, Fig. 2B) were refractory to levetiracetam, phenobarbitone, vigabatrine, and steroids. At the age of 15 months, she showed microcephaly, growth retardation, and severe developmental distortion showing no ability to sit and lacking visual fixation of objects or attention to speech. Multiple audiological screenings by automatic auditory brain response revealed hyperacusis of the left ear.

Besides neurologic symptoms, the patient presented with failure to thrive (Figure S1A‐D), aggravated by repetitive vomiting and swallowing difficulties. At the age of 5 months, she received a gastric/jejunal (PEG‐J) tube for feeding. Gastroesophageal reflux was treated with omeprazole. At the age of 7 months, exocrine pancreas insufficiency was detected with low fecal elastase levels. Pancreatic enzyme replacement therapy (Lipase, Amylase & Protease; Creon^®^) was commenced. In addition, endocrine pancreas insufficiency was suspected with repeatedly low Insulin levels but without clinical signs of diabetes.

A detailed listing of symptoms can be found in Table S1.

### Laboratory findings

Basic laboratory work‐up showed mild hypothyroidism and therapy with L‐thyroxine was started. Routine testing for congenital disorder of glycosylation at the age of 5 months showed negative results. Additionally, reduced levels of serum insulin and C‐peptide were repeatedly measured (three independent measurements performed between 7 and 13 months of age).

### Molecular findings

ES detected approximately 180 Mb of homozygous regions, corresponding to the consanguinity of the parents. Within these stretches, 13 homozygous loss‐of‐function variants were found (Table S2). Since all but one of the identified variants were homozygously present in healthy individuals in the Genome Aggregation Database (gnomAD),^15^ the homozygous nonsense variant in *CCDC186* (NM_018017.2) c.767C>G; p.(Ser256Ter) (hg19, Chr10: g.115910972G>C) was singled out as the sole candidate. Both parents were heterozygous carriers. The variant is predicted to cause a premature termination of translation in exon 4 out of 15 exons and would most likely result in nonsense‐mediated mRNA decay.

### Minimal lifetime risk of *CCDC186*‐associated disease

We assessed the minimal lifetime risk of *CCDC186* related disease by extracting the combined minor allele frequency of reported loss‐of‐function alleles in the gnomAD database and calculating the frequency of biallelic carriers under the assumption of the Hardy–Weinberg equilibrium and mutual independence of the variants as previously described.^16^ The combined minor allele frequency of *CCDC186* alleles was 0.00016, resulting in 1 in 39 million presumingly affected newborns with biallelic loss‐of‐function variants in *CCDC186* (Table S3).

## Discussion

Our female patient presented with failure to thrive, severe developmental disorder and seizures. ES identified a homozygous nonsense variant in the candidate gene *CCDC186* (NM_018017.2: c.767C>G; p.(Ser256Ter)), which is expected to result in nonsense‐mediated mRNA decay. We consider it to be associated with the patient’s phenotype, although (especially in the context of parental consanguinity) other variants contributing to the phenotype cannot be entirely excluded. Within a large sequencing study of 1000 cases from Saudi‐Arabia, a homozygous loss‐of‐function variant in *CCDC186* (NM_018017: c.610C>T; p.(Gln204Ter)) was identified in a patient with failure to thrive, global developmental delay, hypotonia, brain atrophy as well as undescended testis, micropenis, and poor vision.^13^ Thus, a distinct overlap in the reported phenotypes can be observed (Table S1). The pathogenic effect of predicted loss‐of‐function variants in *CCDC186* is further undermined by the absence of homozygous loss of function alleles in healthy individuals in gnomAD, as well as in our in‐house variant database of currently 20,000 exome datasets.

Knockout of *cccp‐1* in *C. elegans* neurons lead to reduced levels of secretory cargo in axonal vesicles^2^ and it was proposed that deficiency of CCDC186 might lead to loss of secretory cargo to the endolysosomal compartment or secretion of incorrectly sorted cargo (Fig. 1).^3^, ^7^ As DCVs are important for axonal and dendritic growth, synaptogenesis, synaptic pruning and myelination,^17^, ^18^, ^19^ the impaired secretion of neuromodulators could result in aberrant neuronal development and impaired synaptic plasticity in patients with a loss of CCDC186. By engaging with the EARP complex, CCDC186 is additionally linked to the endolysosomal pathway. Disruption of the TGN and endolysosomal trafficking due to impaired function of Rab GTPases has further been recognized in neurodegenerative diseases such as amyotrophic lateral sclerosis and frontotemporal dementia.^20^, ^21^

In the affected individuals, truncating *CCDC186* variants additionally lead to severe failure to thrive. While *cccp‐1* knockout in *C. elegans* results in a neurologic phenotype of impaired and slowed locomotion,^2^ homozygous *Otg1/CCDC186* knockout mice show severe postnatal growth retardation and preweaning lethality as well as impaired glucose metabolism with hypoglycemia and low levels of serum insulin.^22^ The critical role of CCDC186 in cargo sorting and insulin secretion upon stimulation was recently confirmed in rat insulinoma cells.^3^ The participation of CCDC186 in the secretion of peptides and hormones in humans might be further strengthened as blood protein levels were found to be associated with a single nucleotide polymorphism in *CCDC186* (rs11595697‐C).^23^ In our patient, blood glucose levels were normal but reduced serum insulin levels as well as C‐peptide levels indicate dysfunctional insulin secretion because of endocrine pancreas insufficiency. Interestingly, our patient also showed additional exocrine pancreas insufficiency, and commencing pancreatic enzyme replacement therapy directly led to weight gain. These findings are suggestive for a pivotal role of *CCDC186* in the pancreas as well, as no genetic alterations in genes associated with chronic pancreatitis were detected by ES. Secondary pancreas insufficiency due to severe gut inflammation or autoinflammation seem unlikely without endoscopic signs nor systemic signs of inflammation. However, it remains unclear whether the reduced secretion of pancreatic hormones and enzymes is a result of dysfunctional DCV secretion alone or if another underlying pathomechanism exists.

Taken together, our findings confirm the association of biallelic loss‐of‐function in *CCDC186* with a phenotype including epileptic encephalopathy and growth retardation. By applying the framework suggested by the Clinical Genome Resource regarding the evaluation of gene‐disease associations,^24^ we recognize there is still limited evidence to support a disease association of *CCDC186*. Thus, further evidence, especially data from experimental studies, is needed to strengthen the evidence level of *CCDC186* as a new disease gene. Whether defects of the endocrine or exocrine systems also contribute to the *CCDC186*‐associated phenotype remains unclear. Additional patients with biallelic variants in *CCDC186* will have to be identified to illuminate the phenotypic spectrum of *CCDC186*‐associated disease.

## Conflict of Interest

The authors report no conflict of interests in relation to the contents of this article.

## Author contributions

MB was responsible for drafting of the manuscript. IB and MW proposed and supervised the manuscript as senior authors. IB, EL, and FBD contributed to clinical data acquisition and provided clinical care to the patient. TB, MB, and MW contributed to the interpretation of genetic data. MB, FBD, EL, IB, and MW contributed to drafting the text and preparing the figures and tables. All authors contributed to the critical revision of the manuscript for intellectual content and gave final approval for the version to be published.

## Supporting information


**Figure S1.** Growth charts of the reported patient.
**Table S1.** Listing of the clinical details of the reported patent with a homozygous loss‐of‐function variant in *CCDC186* in comparison to the previously reported patient by Monies et al. (2017).
**Table S2.** Listing of all identified homozygous loss‐of‐function variants.
**Table S3.** Frequency of heterozygous loss‐of‐function variants in healthy controls and assessed minimal lifetime risk of *CCDC186*‐associated disease.
