# Supplementary material for: A homozygous truncating variant in CCDC186 in an individual with epileptic encephalopathy
Source: Ann Clin Transl Neurol. 2020 Dec 1;8(1):278–83. doi: 10.1002/acn3.51260 (PMC7818090; doi:10.1002/acn3.51260)
Supplement: Supplementary file 1 — Figure S1. Growth charts of the reported patient. Table S1. Listing of the clinical details of the reported patent with a homozygous loss‐of‐function variant in CCDC186 in comparison to the previously reported patient by Monies et al. (2017). Table S2. Listing of all identified homozygous loss‐of‐function variants. Table S3. Frequency of heterozygous loss‐of‐function variants in healthy controls and assessed minimal lifetime risk of CCDC186‐associated disease. [file ACN3-8-278-s001.docx]

**Supplementary material:**

**Supplementary Figure 1**: Growth charts of the reported patient.


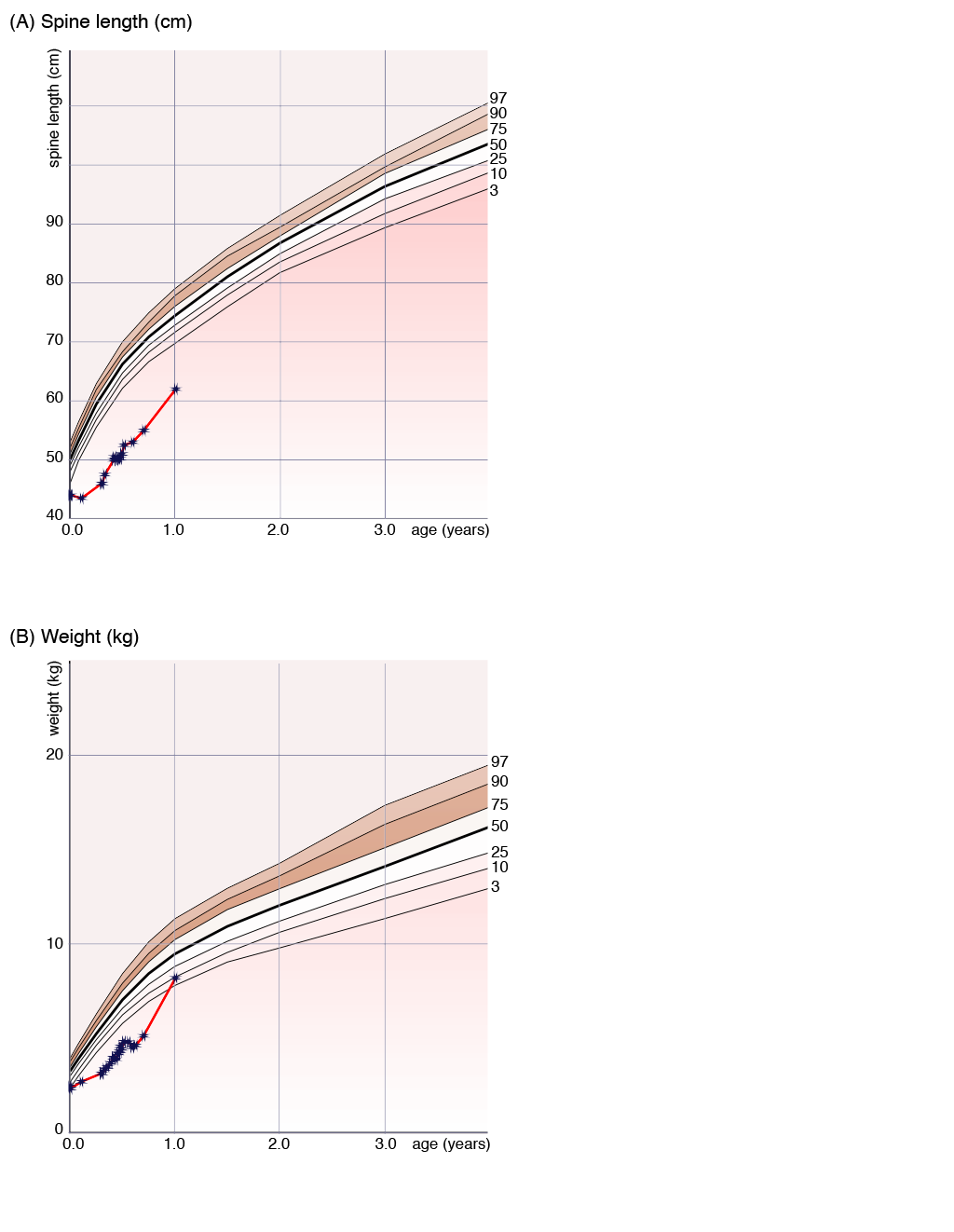

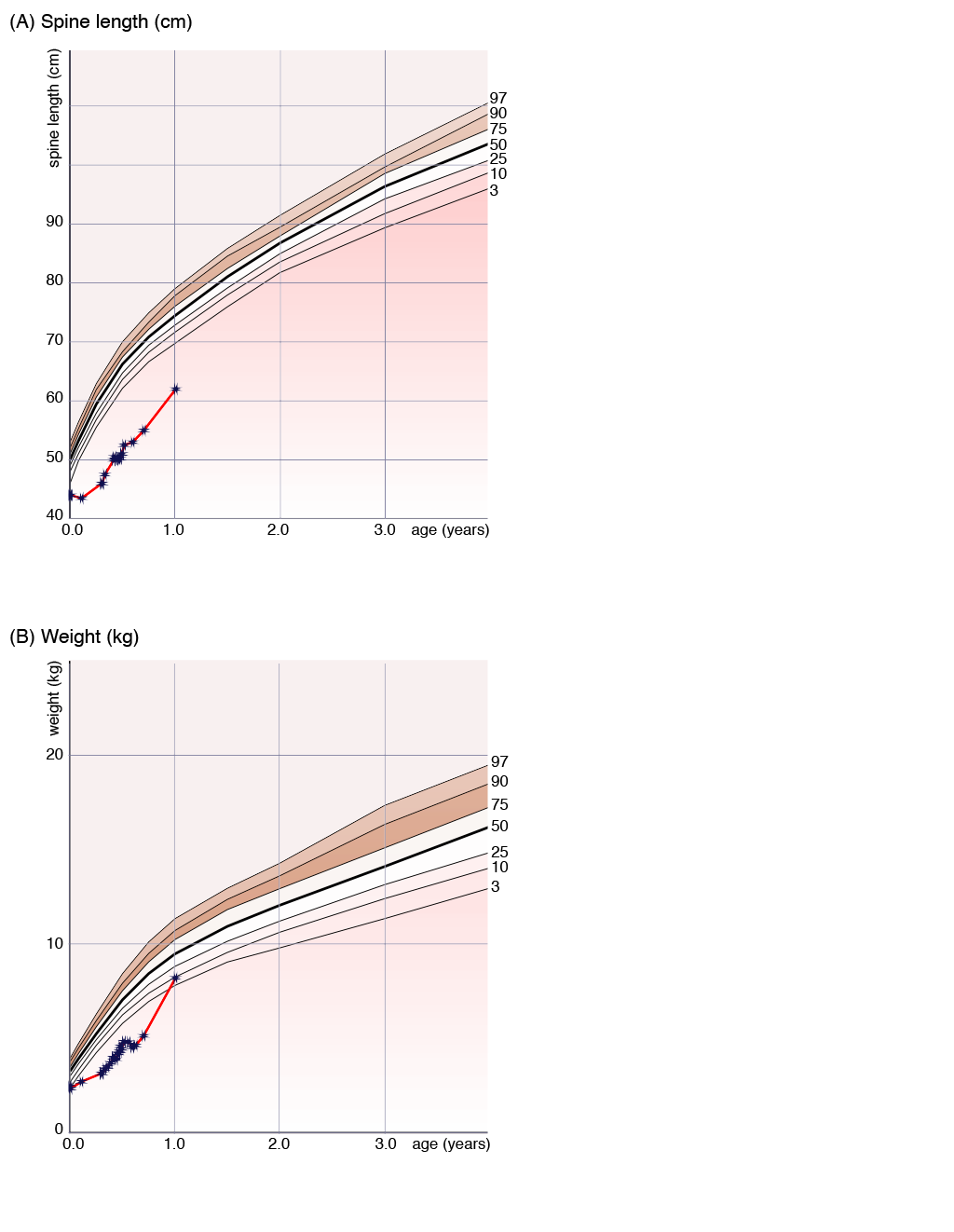

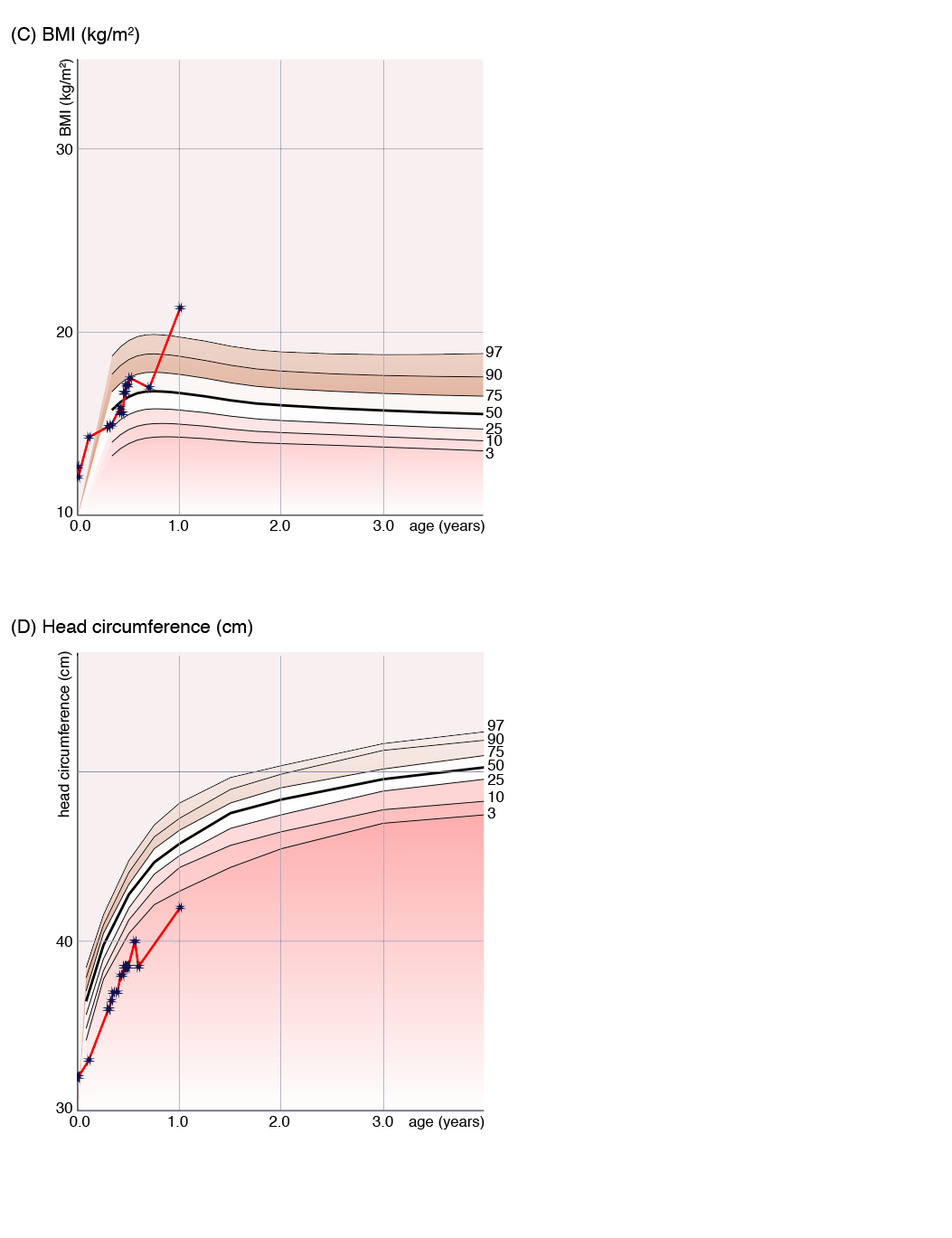

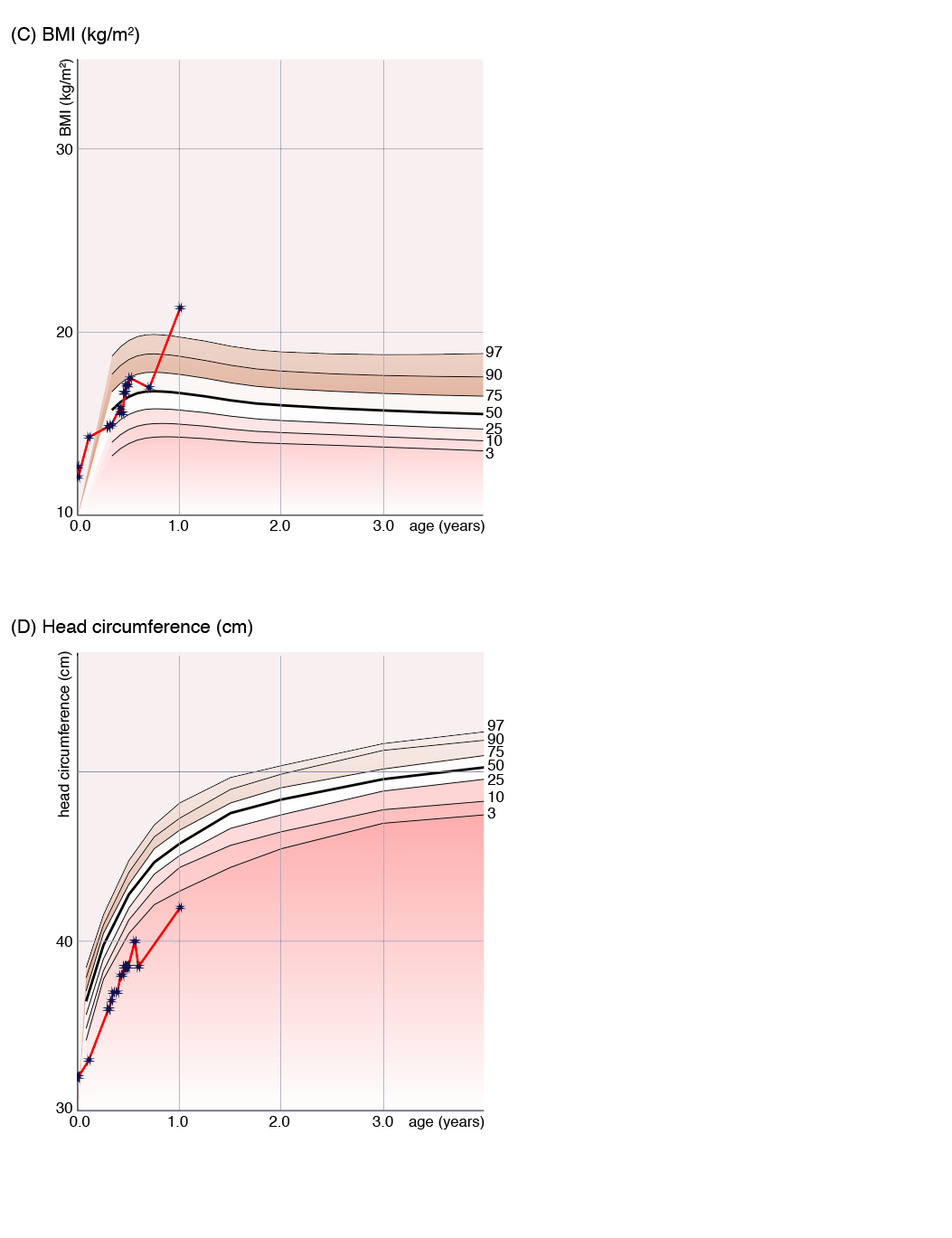


**Supplementary Table 1:** Listing of the clinical details of the reported patent with a homozygous loss-of-function variant in *CCDC186* in comparison to the previously reported patient by Monies et al. (2017).

|  |  | **reported patient** | **Monies et al. (2017)**  **(16W-0208)** |
| --- | --- | --- | --- |
| **Age at last follow-up** |  | 15 months | 4 years |
| **Gender** |  | female | male |
| **Homozygous variants in *CCDC186* (NM_018017.2)** |  | c.767C>G; p.(Ser256Ter) | c.610C>T; p.(Glu204Ter) |
| **Origin** |  | Senegal | Saudi Arabia |
| **Consanguinity** |  | yes | yes |
| **Growth** | **IUGR** | yes (birth weight: 2450g) | NA |
|  | **Failure to thrive** | yes | yes |
|  | **Microcephaly** | yes | NA |
| **Development** | **Developmental delay** | yes (severe) | yes (fine and gross motor delay, intellectual disability) |
|  | **Speech delay** | yes (no speech) | yes |
| **Neurological findings** | **Muscular hypotonia** | yes | yes |
|  | **Seizures** | yes | NA |
| **Congenital anomalies** |  | infundibular pulmonary stenosis | NA |
| **Gastrointestinal findings** |  | yes (vomiting, obstipation, exocrine pancreas insufficiency) | NA |
| **Endocrinologic findings** |  | yes (hypothyreosis, suspected endocrine pancreas insufficiency) | NA |
| **Urogenital findings** |  | no | yes (undescended testis, micropenis) |
| **Ophthalmologic findings** |  | yes (lack of fixation) | yes (poor vision) |
| **Auditory findings** |  | yes (hyperacusis, left side) | NA |
| **Craniofacial features** |  | no distinctive facial features | NA |
| **MRI findings** |  | frontotemporal atrophy | brain atrophy |

**Supplementary Table 2**: Identified homozygous loss of function variants by exome sequencing. Listing of all identified homozygous loss of function variants applying a filter of maximum allele frequency of 0.1% according to the in-house database after removal of incorrectly called variants and intronic variants.

| **Chromosome** | **Gene** | **Transcript** | **cDNA variant** | **Protein variant** | **Omim** | **Class** | **Function** | **Variant alleles** | **SNV Qual** | **Map Qual** | **Depth** | **Percent Var** | **ClinVar** | **gnomAD ea** | **gnomAD aa** |
| --- | --- | --- | --- | --- | --- | --- | --- | --- | --- | --- | --- | --- | --- | --- | --- |
| chr10:115910972-115910972 | CCDC186/ 10orf118 | NM_018017.2 | c.767C>G | p.Ser256Ter | NA | snp | nonsense,noncoding | 2 | 159 | 60 | 44 | 100 |  | NA | NA |
| chr11:5221439-5221442 | OR51V1 | NM_001004760.2 | c.489_492delCTTT | p.Phe163LeufsTer9 | NA | indel | frameshift | 2 | 164 | 50 | 43 | 100 |  | 64115--31--0 | 9577--2542--176 |
| chr12:10569265-10569265 | KLRC3 | NM_002261.2 | c.587+1G>T | ? | NA | snp | splice | 2 | 223 | 49 | 65 | 100 |  | 64304--38--0 | 10166--2172--126 |
| chr12:31256907-31256907 | DDX11 | NM_001257144.1 | c.2853C>A | p.Cys951Ter | 601150 | snp | nonsense,3utr,noncoding | 2 | 282 | 37 | 206 | 99 |  | 47295--17--0 | 8497--565--6 |
| chr12:55945585-55945588 | OR6C4 | NM_001005494.1 | c.575_578delTCTT | p.Leu192GlnfsTer3 | NA | indel | frameshift | 2 | 290 | 55 | 118 | 100 |  | 64202--127--0 | 8383--3714--368 |
| chr13:111335483-111335484 | CARS2 | NM_024537.2 | c.572-3_572-2delCA |  | 612800 | indel | splice | 2 | 207 | 60 | 57 | 100 | Benign | 60450--2--0 | 10959--689--13 |
| chr17:33772713-33772713 | SLFN13 | NM_144682.5 | c.-13-1G>T |  | NA | snp | splice,5utr | 2 | 193 | 60 | 55 | 100 |  | 57331--9--0 | 11396--311--5 |
| chr22:36556768-36556768 | APOL3 | NM_145640.2 | c.172C>T | p.Gln58Ter | NA | snp | nonsense,5utr | 2 | 208 | 59 | 60 | 100 |  | 64525--37--0 | 7609--4229--640 |
| chr2:108443529-108443529 | RGPD4 | NM_182588.2: | c.60delG | p.Ser21ArgfsTer69 | NA | indel | frameshift | 2 | 290 | 48 | 87 | 100 |  | 53336--8--1 | 9668--298--3 |
| chr2:74730314-74730315 | LBX2-AS1 | NM_001009812.1 | c.-329dupG |  | NA | indel | frameshift,5utr,regulation | 2 | 290 | 60 | 89 | 100 |  | 7698--5--0 | 3171--1093--85 |
| chr4:88235097-88235097 | HSD17B13 | NM_001136230.1 | c.465delC | p.Ala156LeufsTer8 | NA | indel | frameshift | 2 | 237 | 59 | 67 | 100 |  | 57303--45--0 | 7171--3456--401 |
| chr8:125568183-125568183 | NDUFB9 | NM_014751.4 | c.1405-38delC |  | 601445 | indel | frameshift,intronic | 2 | 290 | 60 | 210 | 100 |  | 55425--1692--22 | 8396--2317--162 |
| chrX:154290120-154290120 | CMC4 | NM_001018024.2 | c.205T>C | p.Ter69GlnextTer13 | NA | snp | stoploss | 2 | 280 | 60 | 84 | 100 |  | 45293--0--0 | 9332--71--0 |

**Supplementary Table 3:** Frequency of heterozygous loss-of-function variants in healthy controls and assessed minimal lifetime risk of *CCDC186*-associated disease. The table shows all reported loss-of-function variants in *CCDC186* and the respective allele frequency in the gnomAD v2.1.1 dataset.

| **Chromo-some** | **Position** | **rsID** | **Reference** | **Alternate** | **Consequence** | **Protein Consequence** | **Transcript Consequence** | **Annotation** | **Allele Count** | **Allele Number** | **Allele Frequency** |
| --- | --- | --- | --- | --- | --- | --- | --- | --- | --- | --- | --- |
| 10 | 115884906 | rs778829812 | GT | G | p.Thr898ProfsTer17 | p.Thr898ProfsTer17 | c.2692delA | frameshift_variant | 4 | 241146 | 1,6587E-05 |
| 10 | 115884963 | rs756548615 | G | A | p.Gln435Ter | p.Gln435Ter | c.1301C>T | stop_gained | 1 | 248728 | 4,0205E-06 |
| 10 | 115885678 | rs1171341409 | C | CA | p.Leu860PhefsTer27 | p.Leu860PhefsTer27 | c.2579dupT | frameshift_variant | 1 | 251146 | 3,9817E-06 |
| 10 | 115885698 | rs1340695419 | G | A | p.Arg854Ter | p.Arg854Ter | c.2560C>T | stop_gained | 2 | 251178 | 7,9625E-06 |
| 10 | 115885794 | rs1236126404 | CTT | C | p.Lys821SerfsTer5 | p.Lys821SerfsTer5 | c.2462_2463delAA | frameshift_variant | 1 | 251116 | 3,9822E-06 |
| 10 | 115887353 | rs746517320 | GAA | G | p.Phe753SerfsTer5 | p.Phe753SerfsTer5 | c.2258_2259delTT | frameshift_variant | 1 | 251074 | 3,9829E-06 |
| 10 | 115887398 | rs767520724 | G | A | p.Arg739Ter | p.Arg739Ter | c.2215C>T | stop_gained | 1 | 250968 | 3,9846E-06 |
| 10 | 115889649 | rs1421818558 | C | T | c.2182+1G>A | | c.2182+1G>A | splice_donor_variant | 1 | 250920 | 3,9853E-06 |
| 10 | 115889728 | rs753516890 | G | A | p.Arg702Ter | p.Arg702Ter | c.2104C>T | stop_gained | 2 | 281766 | 7,0981E-06 |
| 10 | 115891065 | rs199821888 | G | A | p.Arg648Ter | p.Arg648Ter | c.1942C>T | stop_gained | 1 | 228274 | 4,3807E-06 |
| 10 | 115894670 | rs1361963132 | A | G | c.1655+2T>C |  | c.1655+2T>C | splice_donor_variant | 1 | 240854 | 4,1519E-06 |
| 10 | 115894775 | rs758001809 | C | A | p.Glu518Ter | p.Glu518Ter | c.1552G>T | stop_gained | 1 | 248372 | 4,0262E-06 |
| 10 | 115894793 | rs776927562 | G | A | p.Arg512Ter | p.Arg512Ter | c.1534C>T | stop_gained | 1 | 244868 | 4,0838E-06 |
| 10 | 115894810 | rs1376612258 | T | TTC | p.Lys506ArgfsTer4 | p.Lys506ArgfsTer4 | c.1515_1516dupGA | frameshift_variant | 1 | 242078 | 4,1309E-06 |
| 10 | 115895905 | rs761037441 | A | T | c.1425+2T>A |  | c.1425+2T>A | splice_donor_variant | 2 | 250202 | 7,9935E-06 |
| 10 | 115895936 | rs1419473942 | G | A | p.Gln466Ter | p.Gln466Ter | c.1396C>T | stop_gained | 1 | 250558 | 3,9911E-06 |
| 10 | 115896006 | rs1466763519 | C | A | c.1327-1G>T |  | c.1327-1G>T | splice_acceptor_variant | 1 | 245720 | 4,0697E-06 |
| 10 | 115896007 | rs755392771 | T | C | c.1327-2A>G |  | c.1327-2A>G | splice_acceptor_variant | 1 | 245574 | 4,0721E-06 |
| 10 | 115896980 | rs781536227 | G | A | p.Arg431Ter | p.Arg431Ter | c.1291C>T | stop_gained | 1 | 251026 | 3,9837E-06 |
| 10 | 115904306 | rs750455162 | T | A | p.Lys391Ter | p.Lys391Ter | c.1171A>T | stop_gained | 1 | 251158 | 3,9816E-06 |
| 10 | 115910864 | rs760354548 | T | TG | p.Gln292ProfsTer11 | p.Gln292ProfsTer11 | c.874dupC | frameshift_variant | 1 | 250548 | 3,9913E-06 |
| 10 | 115910936 |  | GAAGCTTTAACTTCTTTATTAAGTTCTTCTATTCTTGATTCTAACTGTAAAGAAAATTATTTCCAAATTATTTTTATAGCTAT | G | c.760-39_802delATAGCTATAAAAATAATTTGGAAATAATTTTCTTTACAGTTAGAATCAAGAATAGAAGAACTTAATAAAGAAGTTAAAGCTT | | c.760-39_802delATAGCTATAAAAATAATTTGGAAATAATTTTCTTTACAGTTAGAATCAAGAATAGAAGAACTTAATAAAGAAGTTAAAGCTT | splice_acceptor_variant | 2 | 250516 | 7,9835E-06 |
| 10 | 115917316 | rs759642671 | TTTAA | T | p.Ile251AsnfsTer3 | p.Ile251AsnfsTer3 | c.752_755delTTAA | frameshift_variant | 2 | 238150 | 8,3981E-06 |
| 10 | 115922394 | rs763932967 | ACT | A | c.632_632+1delAG | | c.632_632+1delAG | splice_donor_variant | 1 | 195750 | 5,1086E-06 |
| 10 | 115922493 | rs765734254 | G | A | p.Arg179Ter | p.Arg179Ter | c.535C>T | stop_gained | 2 | 251098 | 7,9650E-06 |
| 10 | 115922660 | rs781063741 | C | CTTAA | p.Arg123IlefsTer11 | p.Arg123IlefsTer11 | c.364_367dupTTAA | frameshift_variant | 4 | 282046 | 1,4182E-05 |
| 10 | 115922695 | rs1436133199 | TGTTTC | T | p.Glu110ArgfsTer21 | p.Glu110ArgfsTer21 | c.328_332delGAAAC | frameshift_variant | 1 | 249506 | 4,0079E-06 |
| 10 | 115922958 |  | CCTTT | C | p.Glu24ThrfsTer14 | p.Glu24ThrfsTer14 | c.66_69delAAAG | frameshift_variant | 1 | 251158 | 3,9816E-06 |
|  |  |  |  |  |  |  |  |  | **Combined minor allele frequency** | | 1,60E-04 |
|  |  |  |  |  |  |  |  |  | **minimal lifetime risk** | | 2,5622E-08 |

**Supplementary Figure Legends:**

**Supplementary Figure 1:** Growth charts of the reported patient. Black asterisks connected by a red line mark measurements of the reported patient. Percentile curves (3rd/10th/25th/50th/75th/90th/97th percentiles) show the age dependent distribution of the respective body measurement. (A): Spine length measured in cm. (B): Body weight measured in kg. (C): BMI (kg/m^2^). (D): Head circumference measured in cm.

**Supplementary Table 1:** Listing of the clinical details of the reported patient with a homozygous loss-of-function variant in *CCDC186* in comparison to the previously reported patient by Monies et al. (2017). IUGR: intrauterine growth retardation; MRI: magnetic resonance imaging; NA: not applicable.

**Supplementary Table 2:** Listing of all identified homozygous loss-of-function variants by exome sequencing applying a filter of maximum allele frequency of 0.1% according to the in-house database after removal of incorrectly called variants and intronic variants.

**Supplementary Table 3:** Frequency of heterozygous loss-of-function variants in healthy controls and assessed minimal lifetime risk of *CCDC186*-associated disease. The table shows all reported loss-of-function variants in *CCDC186* and the respective allele frequency in the gnomAD v2.1.1 dataset.
